# Financial markets’ deterministic aspects modeled by a low-dimensional equation

**DOI:** 10.1038/s41598-022-05765-z

**Published:** 2022-02-01

**Authors:** Giuseppe Orlando, Michele Bufalo, Ruedi Stoop

**Affiliations:** 1 Department of Economics and Finance, University of Bari, 70124 Bari, Italy; 2 Department of Methods and Models for Economics, Territory and Finance, University of Rome “La Sapienza”, 00185 Rome, Italy; 3 Department of Physics and of Neuroinformatics, University and ETH of Zürich, 8057 Zurich, Switzerland

**Keywords:** Physics, Applied physics, Information theory and computation, Statistical physics, thermodynamics and nonlinear dynamics, Mathematics and computing, Applied mathematics, Statistics

## Abstract

We ask whether empirical finance market data (Financial Stress Index, swap and equity, emerging and developed, corporate and government, short and long maturity), with their recently observed alternations between calm periods and financial turmoil, could be described by a low-dimensional deterministic model, or whether this requests a stochastic approach. We find that a deterministic model performs at least as well as one of the best stochastic models, but may offer additional insight into the essential mechanisms that drive financial markets.

## Introduction

Substantial mathematically minded economic research has dealt with the question whether financial data contain in a substantial manner low-dimensional chaotic features, or whether their nature requires the stochastic approach predominantly used by the practitioners dealing with the analysis of financial markets. A comprehensive survey of the various methods that have been used, and the conclusions they have arrived at, has recently been published^1^. In that survey and more generally, the approaches advocating a chaotic nature of the data have almost exclusively relied on indirect statistical quantifiers, such as evaluation of Lyapunov exponents and fractal dimensions^2,3^, data recurrence quantification analysis^4^, or BDS-testing^5^. While such methods may suggest the presence of a chaotic process in the data, an ultimate test by comparing directly financial time series with their stochastic and with their low-dimensional deterministic modeling, is still missing. We will provide here such a comparison.

In theoretical studies, financial time series have predominantly been seen as pure jump processes^6,7^, drift-diffusion models^8–10^, jump-diffusion models that combine the former two^9,10^, or as stochastic volatility models^11,12^. Many of these models are built on the assumption of $\alpha$-stable distributions having finite moments to the order needed, or on distributions truncated to have such properties. In physics, the connection between diffusion and maps and their generated distributions has been a prominent theme^13–18^. Recently, wavelet approaches were used as well^19,20^, as wavelets are a convenient tool for, e.g., distinguishing activity jumps from noise^21,22^.

A major goal in practical econometrics is the prediction of the mean and the volatility of markets. A standard stochastics-based tool for this is the ARIMA-GARCH procedure^23^, where the ARIMA part of the approach models and forecasts the first moment (the mean), and the GARCH part the second moment (the volatility) of the process. Recent dynamics of financial markets have shown spikes both in volatility and in performance (emerging from the impact of COVID-19 in a black swan event^24^), effects that are difficult to deal with. Such phenomena, however, exhibit a strong affinity with the kind of data commonly recorded in neuroscience from cortical neurons, where the neuron’s activity is the result of many single (excitatory or inhibitory) synaptic stimulations^25^. The neuron’s response can be seen as the result from impinging input that can, in a generalized sense, seen to be coupled by the neuron’s strongly nonlinear internal machinery. Generally, the produced nonlinear effects would be difficult to predict; in the case of a neuron they can, however, be described by a simple two-dimensional deterministic Rulkov-map^26^. This map reproduces the whole spectrum from simple to very complex behavior of neurons^27^, including effects similar to what we see in financial data. For applying this picture to the finance markets, relaying buying-selling activities towards the observed collective financial market behavior, the eco-societal environment would act as the coupling element.

Using this perspective, we will directly compare financial market data with the results from a modified Rulkov-map approach, and, as a competitor, with the commonly used optimized ARIMA-GARCH model, demonstrating that the modeling results only differ by what a small variation of white Gaussian noise would trigger. In addition to this, the map-based description offers advantages in terms of conceptual simplicity and computational speed (cf. “Rulkov-map modeling of the Financial stress index” and “Conclusions” sections). In particular, an obvious asset of the deterministic model is that it operates in a low-dimensional subspace of models defined by means of the form of the deterministic equation, whereas the stochastic approach lacks such a limiting element. Whereas the ARIMA-GARCH approach deals separately with mean and volatility, in our two-dimensional deterministic approach, the first vector component represents an instantaneous activity that is functionally linked to an underlying trend represented by the second vector component. With this, the Rulkov-map equation offers insight into how the driving sub-processes are entwined and to what effects, e.g., a change of one of its few parameters may lead. By this, our explicit deterministic approach opens a new perspective towards understanding financial markets. In what follows, we present a description of our novel modeling approach, followed by review of the classical modeling tools that we have to compare with. Then, we will recall the most popular measures for model comparison, before we display and finally discuss the results obtained, including the performance obtained from two more recent models. Our main focus will, however, not be that of a competition between the stochastic vs. deterministic approaches, but to show that there is a substantial deterministic part in the data that could be exploited for the explanation and prediction of financial market data.

## Modeling tools

### Generalized Rulkov map

During the recent turbulent years, ten Asian emerging stock markets showed non-linear serial dependence, and in real exchange rates of developing and emerging market economies^28^, in four USA stock market indices^29^, and in USA short rate indices^30^, mean reversion was observed; jumps are generally common in financial time series^31^. Similarly, biological neurons exhibit a two-phase response: an activation phase, characterized by a transient and presence of fast oscillations, and a subsequent plateau phase characterized by more regular oscillations. The two-dimensional Rulkov map family of biological neurons^26^ combines a one-dimensional fast subsystem (*x*) and a one-dimensional slow subsystem (*y*), where the latency to a stimulating event depends on the fast-slow dynamics. While this interplay between fast-slow dynamics is a general mechanism unterlying pattern formation, the Rulkov map offers through the involved parameters the possibility to adapt the general mechanism to specific features exhibited by a process modeled. In particular by its recursive nonlinear and mean reverting properties, Rulkov maps may be expected to be highly suitable for the modeling of financial  time series, in particular regarding the occurrence of data clusters, heteroskedasticity, mutual synchronization, chaos and regularization of bursts of activity across the markets, after-shock asset classes, and more^32^. In our application, we will optimize the Rulkov family model to best reproduce the processes and events observed in our data.

To provide a first general insight into the nature of Rulkov maps, we start from a two-dimensional non-linear recurrence of the form

1 $$\begin{aligned} x_{t+1} = f_\alpha (x_t) + \gamma \, y_t + \delta , \, \text{ with } \, f_\alpha (x_t) = \dfrac{\alpha }{1+x_t^{n}}, \qquad n \in {\mathbb {N}}, \, \alpha , \gamma , \delta , x\in {{\mathbb {R}}}. \end{aligned}$$

Such a setting is widely used for the modelling short-time effects like pulses and shocks in time series. Vector component *y* captures a moving average trend implemented as

2 $$\begin{aligned} y_{t+1} = \beta \, y_t - \mu \, x_{t} + \eta ,\text{ with } \, \beta , \mu , \eta , y \in {{\mathbb {R}}}. \end{aligned}$$

Combining Eq. (1) with Eq. (2) leads to

3 $$\begin{aligned} x_{t+1}= & {} f_\alpha (x_t) + \gamma \, y_t + \delta , \nonumber \\ y_{t+1}= & {} \beta \, y_t - \mu \, x_{t} + \eta . \end{aligned}$$

This version, that differs slightly from the original Rulkov version, is the map that we will use for our financial time series modeling; its two-dimensional form takes equally care of the short-term (*x*) and the average prediction (*y*) horizon (the two main subjects of interest in financial modeling), which provides the basis for an optimal modeling framework. Of importance are the following characteristics hosted by function $f_{\alpha }$.

#### **Proposition 2.1**

*For any (even) integer* *n*, *the function* $f_{\alpha }(x)$*, defined in* (1)*, satisfies:*

(i) *If* $\alpha >0$ *then* $f_{\alpha }(x)$ *assumes its global maximum value (*$\alpha$*) at* $x=0$*; otherwise,* $f_{\alpha }(x)$ *assumes its global minimum value (*$\alpha$*) at* $x=0$
(ii) *For* $x\rightarrow \pm \infty$, $f_{\alpha }(x)$ *approaches 0*
(iii) $\lim _{n\rightarrow +\infty } f_{\alpha }(x)=\alpha \Pi \biggl (\frac{x}{2}\biggr )$*, where* $\Pi \biggl (\frac{x}{2}\biggr )=0\,\,\,\, \text{ for }\,\,\,|x| > 1, \, \,\, \text{ and } \,\,\, \Pi \biggl (\frac{x}{2}\biggr )=1\,\,\, \text{ for }\;\,\,\,|x|\le 1$ *(the rectangle function)*

#### *Proof*

Without loss of generality we set $\alpha >0$

(i) $f'_{\alpha }(x)=-\frac{n \alpha x^{n-1}}{(1+x^n)^2}$, for any $x\in {{\mathbb {R}}}$. This equals zero only for $x=0$, is larger than zero for $x<0$ and smaller than zero for $x>0$. Hence, $x=0$ is a global maximum for $f_{\alpha }$
(ii) is immediate
(iii) is the consequence of $\lim _{n\rightarrow +\infty } \frac{1}{1+(2x)^{n}}=\Pi (x)$, for any even integer *n*, $x\in {{\mathbb {R}}}$

$$\square$$

**Figure 1:**
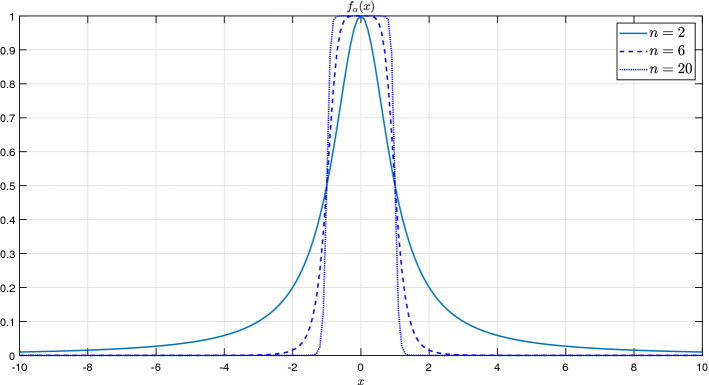
$f_\alpha (x)$ for different values of *n*, using $\alpha =1$.

Figure 1 exhibits that the choice of $n=2$ provides the *x*-sensitivity that according to our tests is desirable; this will be our choice in the following, unless specified otherwise.

For our demonstrations, we will mainly use *single* Rulkov maps; to simulate the interaction between markets, asset classes, and more, Rulkov maps labelled by indices *i* and *j* were *coupled* according to $\{x_{t+1}^{(i,j)} = f_{\alpha ^{(i)}}(x_t^{(i)}) + \gamma ^{(i)} \, y_t^{(i)} + \delta _{t-\tau ^{(i)}}^{(i,j)}, \,\, y_{t+1}^{(i)} = \beta ^{(i)} \, y_t^{(i)} - \mu ^{(i)} \, x_{t}^{(i)} + \eta ^{(i)} \}$, where the coupling $\delta _{t-\tau ^{(i)}}^{(i,j)} = \dfrac{1}{1+\exp (-\kappa ^{(i)} x_{t-\tau ^{(i)}}^{(j)} - \theta ^{(i)})}$ describes a time-lagged interaction between the time series *i* and *j* (and likewise for interchanged indices). The idea underlying this specific coupling is that such an interaction will mostly occur on a short time-scale. $\alpha , \beta , \gamma , \mu , \eta , \delta , \kappa , \theta$ are system parameters, whereas $\tau$ coincides with the time-series specific embedding dimension used for the reconstruction of the phase-space from the one-dimensional time series^2,33^. If a single time series is our object of study, we will use the term specification “single”; if we consider the coupling among two time series, we will use the term specification “coupled” to indicate this.

For our applications of the Rulkov map, the involved parameters must be chosen appropriately. For this, we extract the *x* and *y* initial conditions from the beginning of the data (for details see “Forecasting” section), then calibrate the parameters by running over the rest of the data a robust nonlinear least squares regression, using an iteratively re-weighted least squares algorithm that, at each iteration, recomputes the weights based on the residual from the previous iteration. This process progressively continues until the weights converge. For details see Holland et al.^34^.

### ARIMA-GARCH

To assure validity, we will mainly benchmark our optimized deterministic Rulkov-map approach against an optimized ARIMA-GARCH model (consisting of optimized Auto-Regressive Integrated Moving Average model (ARIMA) combined with an optimized Autoregressive Conditional Heteroskedasticity model (GARCH)). Probably the currently most used and best-documented stochastic approach^23^. A less detailed comparison to the performance of more advanced versions of the stochastic approach is furnished in the context of the conclusions from this comparison.

In financial data, potential serial correlations deserve special attention. Their effects can be eliminated by differencing the original non-stationary time series once, or if required, a higher number of times (the procedure must, however, not be pushed too far, as higher $(> 2)$ orders tend to distort the desired frequency components^35^). In our tests up to order four, only the first order yielded improvements. The resulting stationary time series can then be treated by the Box-Jenkins methodology (ARMA or ARIMA)^23,36^.

The AR part of ARIMA combines a linear extrapolation based on past observations and the MA part contributes the influence by past errors. In short note, for the optimal ARIMA-model, the equation $(1-\sum _{i=1}^p \varphi _i L^{i})(1-L)^d x_t=(1+\sum _{i=1}^q \theta _i L^{i}) \varepsilon _t$ is solved, where *x* denotes the data values, $\varepsilon$ the errors (white noise), *L* the time-shift operator needed for a *d*-fold differencing towards stationary signals, *p* the lag-level considered and *q* the order of the moving-average model. $\varphi _i$ are the linear autoregressive coefficients and $\theta _i$ the moving average coefficients that apply to past errors. From this, the GARCH part then evaluates the variance of the process conditioned on the observed past data values and variances. The ARIMA(p,d,q)-model providing the optimal description of the data can then be used for forecasting. For example, given an ARIMA(2,1,0)-model, we obtain $X_t=\mu +(\varphi _1 (x_{t-1} - \mu )) + (\varphi _2 (x_{t-2} -\mu )) + \varepsilon _t$ for the stationary time series, where $\mu$ is the mean of time series $x_t$ (expected to be small), and $\varepsilon _t$ is white noise term with zero mean and constant variance. The GARCH part of the approach estimates the volatility based on a convex linear combination of its past values, its long-average persistence term, and the exponentially weighted moving average on past data; GARCH(a,b) models take care of contributions that lag further back in time *a* with respect to the generating time series, *b* with respect to the past variances. The determination of the $\{p,d,q\}$ and $\{a,b\}$ polynomials is made by using the Bayesian information (BIC) and the Akaike information (AIC) criteria^37^. The parameter-optimized ARIMA-GARCH model will be denoted in the following by ARIMA-GARCH$^*$. When in the following we mention the *calibration* of an algorithm, we mean that its parameters are optimized over the entire or a part of the data.

### Forecasting

A forecasting process consists of multiple estimations over a rolling window, i.e, in distinction to the parameter calibration over the full data set, we calibrate the ARIMA-GARCH and the Rulkov map on a window, or partition, of the data set. Given our weekly data, we take the full time series and consider only the first 52 data points (i.e., a trading year). We extract over that partition the best ARIMA-GARCH or Rulkov model, and we forecast the next value (i.e., the 53rd data point). In the next step, we consider the interval starting with the second data point to the 53rd data point, and redo all the calibration; with the calibrated model we obtain a new forecast for the 54th data point, etc. For the ARIMA-GARCH process, this involves two calibrations (for the *x* and the *y* separately). For the Rulkov map, this must be only done once.

A standard cost-effective zero-level benchmark against which more sophisticated model predictions can be compared is the naïve model^36^. This model forecasts a value equal to last period’s observed values, resulting in a *in-sample one step ahead* algorithm. A time series ${\hat{x}}_{N+t}=x_N (t \ge 1)$

$X = \lbrace x_h: h \in [1\,\,N] \rbrace$, is forecasted time *t* ahead as ${\hat{x}}_{N+t}=x_N \,\, (t \ge 1)$, which produces an error of size $\dfrac{1}{N-1} \sum _{h=2}^{N} \left| x_h - x_{h-1} \right|$.

### Elements of model results comparison

To compare modeling approaches, we use a set of metrics:

#### Normalized square root mean error

The root mean squared error (RMSE) defined as

$RMSE=\sqrt{\frac{1}{N}\sum _{h=1}^N e^2_h}$, measures the accuracy of a model in terms of goodness of fit, where $e_h$ denote the residuals between the observations and the simulations over *N* time steps. Hence, the values below unity indicate a good fit. RMSE, however, depends on the scale of the observed data and is, by this virtue, outlier-sensitive. For this reason, we will apply the normalized root mean squared error (NRMSE):

4 $$\begin{aligned} NRMSE= \dfrac{RMSE}{x_{\max } -x_{\min }}, \end{aligned}$$

where $x_{\max }$ denotes the maximum value and $x_{\min }$ the minimum value of the observed sample data.

#### Relative mean absolute error

The relative mean absolute error is

5 $$\begin{aligned} RMAE = \dfrac{MAE^{(1)}}{MAE^{(2)}}, \end{aligned}$$

where $MAE^{(1)}$ is the mean absolute error of the model of interest and $MAE^{(2)}$ is the error of some benchmark model^38^. A value greater than unity indicates that the chosen model performs worse than the benchmark.

#### Mean absolute percentage error

To measure the prediction quality of a model, often the mean absolute percentage error (MAPE)

6 $$\begin{aligned} MAPE=\frac{1}{N}\sum _{h=1}^N \biggl | \frac{e_h}{x_h}\biggr | \end{aligned}$$

is used, where $e_h$ denotes the residuals between the observations $x_h$ and their simulations over *N* time steps^39^. Table 1 exhibits the generally accepted MAPE accuracy levels^40^.

**Table 1:** MAPE interpretation guidelines.

MAPE	
$<10\%$	Highly accurate forecasting
$10{-}20\%$	Good forecasting
$20{-}50\%$	Reasonable forecasting
$>50\%$	Inaccurate forecasting

#### Dynamic time warping distance

**Figure 2:**
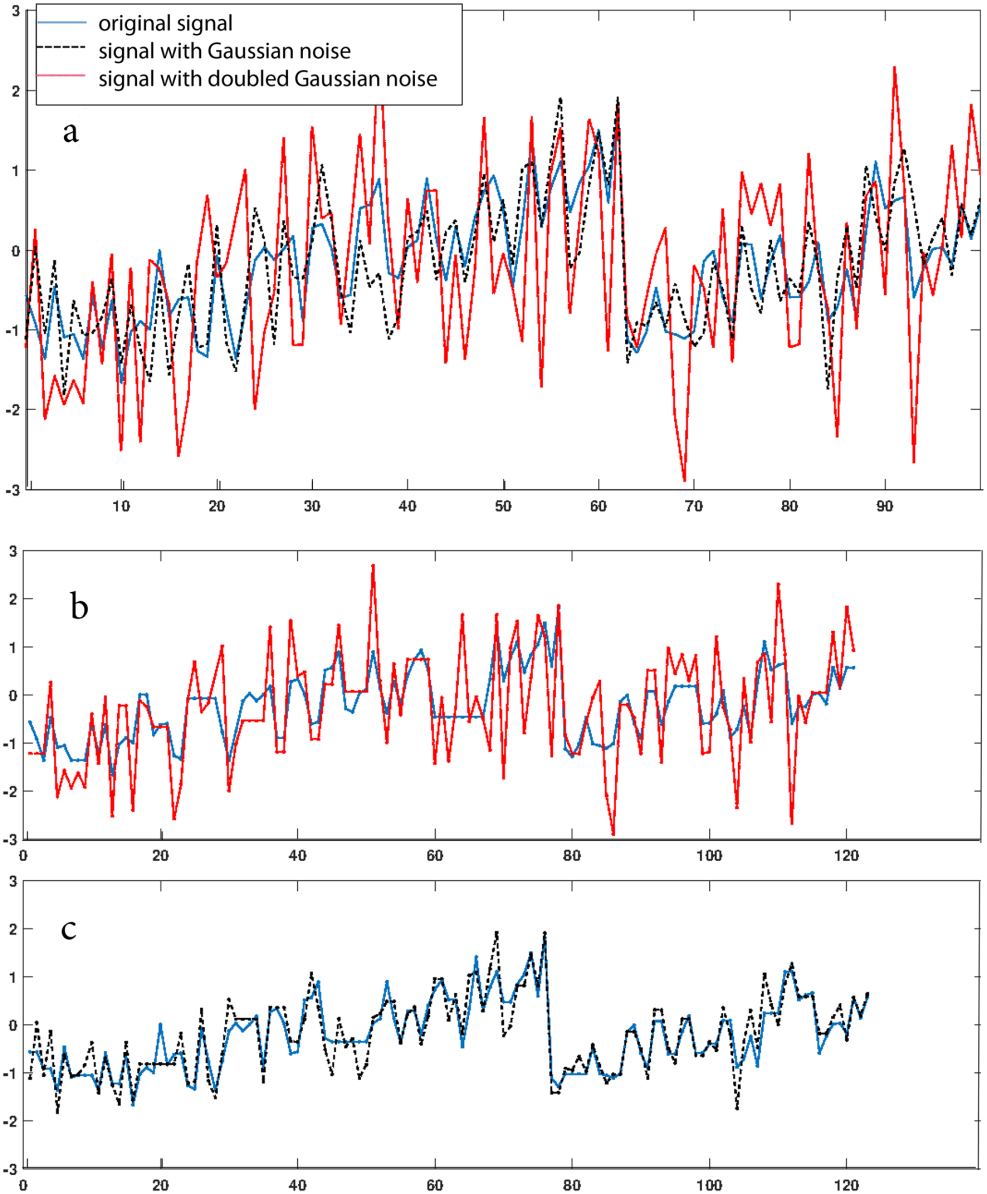
Calibration of DTWD effects: (**a**) Perturbed sawtooth wave (blue), the same upon the addition of white Gaussian noise (black, $\sigma =1/2$) and, finally, of white Gaussian noise of doubled strength (red, $\sigma =1$). (**b**) and (**c**) Signals after DTWD application (same coloring).

To scrutinize how close models are, dynamic time warping distance (DTWD) can be used. This technique finds the optimal alignment between two time series by stretching or shrinking on time series along the time axis, and then evaluates the Euclidean distance between the two^41,42^. This commonly used technique in finance and econometrics^32,43,44^ copes with slightly temporally varying responses in one of the time series^45^. This feature has made DTWD the predominant tool in speech recognition (for determining whether two waveforms represent the same spoken phrase) and one of the standard methods used for classification problems in data mining^46,47^. In our application, DTWD will include information on how much the predicted time series is shifted against the data modeled. In the comparison between different modelings, such effects may play a role, but are expected to be close to the ordinary Euclidean distance between time series. DTWD-results obtained for the artificial system displayed in Fig. 2 will later be used to assess how strong distinct modeling approaches of financial market data differ.

## Data investigated

In Table 2 we provide an overview of the empirical financial time series used for our numerical evaluations. Whereas common standard dataset abbreviations will be used otherwise, the abbreviation *BAMLEM* will refer to the *Emerging Markets Corporate Plus Index Total Return Index Value*.

**Table 2:** Datasets used: (i) The Financial Stress Index STLFSI2^48^ measures the degree of financial stress in the markets.

#	Info provider code	Description	Asset class/issuer	Market
i	STLFSI2	Financial Stress Index	Composite Index	Developed
ii	SWAPS1Y3M	USD Basis Swap 1Mv3M	Swap	Developed
iii	SPX	S&P 500	Equity	Developed
iv	IBOV	Bovespa	Equity	Emerging
v	BAMLEM	AAA-A Em. Mkt Corp TR	Bond Corporate	Emerging
vi	DGS10	10-Y Treasury Const. Mty	Bond Government	Developed

It is constructed from 18 weekly data series, all of which are weekly averages of daily data series: Seven interest rates, six yield spreads, and five other indicators that each captures some aspect of financial stress. Time frame: 31 December 1993–27 November 2020 (ii) USD Basis Swap 1Mv3M returns which is a swapping 1 year versus 3 months, Time frame: 17 January 1986–29 May 2020 (iii) S&P 500 index returns, Time frame: 30 December 1927–29 May 2020 (iv) Bovespa index returns, Time frame: 05 January 1990–29 May 2020 (v) ICE BofA AAA-A Emerging Markets Corporate Plus Index Total Return Index Value [BAMLEM], Time frame: 08 January 1999–29 May 2020 (vi) 10-Year Treasury Constant Maturity Rate [DGS10], Time frame: 08 January 1962–25 May 2020.

## Rulkov-map modeling of the financial stress index

In finance, the fast *x* and the slow *y* components correspond to the actual data and their memory, respectively, where the latter is approximated by the exponentially weighted moving average (EWMA). Since the two components follow well-separated time scales, this suggests to characterize the instability of each component individually by means of a one-dimensional Lyapunov exponent, although the underlying system is in fact two-dimensional.

**Figure 3:**
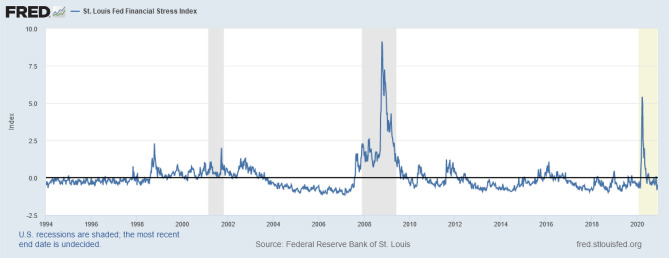
Financial stress index STLFSI2. USA recessions correspond to shaded areas. (Source: FRED^48^).

The Financial Stress Index STLFSI2^48^, see Fig. 3, will be our primary focus in financial data modeling. It is obtained from seven interest rates, six yield spreads, and five other indicators by means of principal component analysis (PCA), as each of these variables captures aspects of financial stress. Accordingly, whenever the level of financial stress in the economy changes, the data series are expected to change in a coherent manner. Positive stress values indicate higher-than-average stress in financial markets, while negative values indicate lower-than-average levels of stress. Our financial stress data covers the 2008–2009 financial crisis, as well as the ongoing turmoil in financial markets stemming from the fear and uncertainty associated with the COVID-19 pandemic. As the best ARIMA-GARCH models in terms of Bayesian information criterion (BIC) and Akaike information criterion (AIC) were those integrated of order one^36^, we calibrated all models investigated (Rulkov map, Naive model and the optimized ARIMA-GARCH model) over the first differences of the STLFSI2.

**Figure 4:**
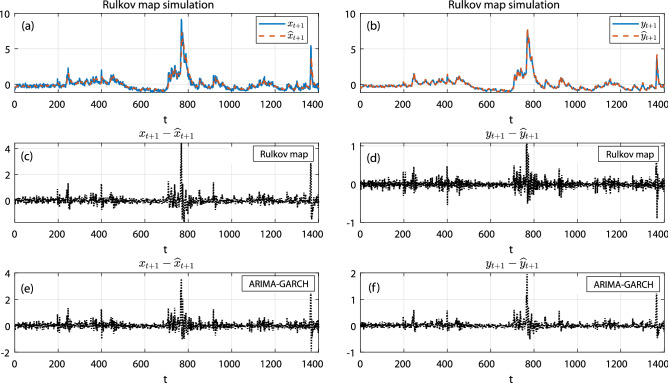
(**a**) Rulkov variable ${\hat{x}}$ (solid blue line) and STLFSI2’s immediate value *x*, dashed red line); (**b**) Rulkov variable ${\hat{y}}$ (solid blue line) and STLFSI2’s long term value *y* (approximated the moving average over $m=52$ data points of *x*, dashed red line). (**c**) Difference between Rulkov ${\hat{x}}$-value and STLFSI2’s value *x*; (**d**) Difference between Rulkov’s ${\hat{y}}$ and STLFSI2’s long term value *y*. (**e**) Difference between ${\hat{x}}$-variable of the calibrated ARIMA-GARCH$^*$ and the immediate value of STLFSI2 *x*; (**f**) Difference between the ${\hat{y}}$-variable of the calibrated ARIMA-GARCH$^*$ and STLFSI2’s long term value *y*.

Below we report the accuracy of data modeling achieved by our Rulkov map approach, then the latter’s potential for making forecasts, and we finally present evidence of chaotic behavior in the data. For the Rulkov map, an $\{x,y\}$-pair provided the input, from which a corresponding output $\{{\hat{x}}, {\hat{y}}\}$ was generated, where for with every *x*-value, an exponential weighted STLFSI2 moving average yielded the corresponding *y*-value. In the stochastic approach, after the two optimized ARIMA-GARCH$^*$ models were determined (one for the *x* and one for the *y*-coordinate), similarly each $\{x,y\}$-pair was fed into the model, generating a corresponding output $\{{\hat{x}}, {\hat{y}}\}$ (for model details, cf. “ARIMA-GARCH”). In Fig. 4 top row, we show how the Rulkov map given the properly calibrated parameters of Table 3, replicates the bursting dynamics of STLFSI2. In the second row, we display the measured error and, in the third row, we compare this to the one obtained from a calibrated ARIMA-GARCH$^*$ model (where the properly calibrated parameters are given in Table 4). The greater simplicity of the model with substantial difference in the number of involved parameter, leads to a speed-up of the Rulkov calibration process by a factor of 20, compared to the ARIMA-GARCH calibration process.

**Table 3:** Parameters of the Rulkov map used for the STLFSI2 data set.

Time series	$\alpha$	$\gamma$	$\delta$	$\beta$	$\mu$	$\eta$
STLFSI2	0.1946	0.9251	− 0.1512	0.5770	0.4167	0.0003

**Table 4:** ARIMA-GARCH* (2,1,2)-(1,1) coefficients for variable *x* and ARIMA-GARCH* (2,1,1)-(2,1) coefficients for variable *y*, listed according to the AR-I-MA-G-ARCH parts of the algorithm (where two of the constants appearing in the ARIMA as well as in the GARCH parts have been added, leading to 16 (instead of 20) parameters to be listed).

Time series	Variable	AR{1}	AR{2}	MA{1}	MA{2}	Const.	G{1}	ARCH{1}	Const.
STLFSI2	*x*	0.0553	0.8534	− 0.1152	− 0.8784	0.0007	0.9942	0.3250	− 0.0309

Time series	Variable	AR{1}	AR{2}	MA{1}	Const.	G{1}	G{2}	ARCH{1}	Const.
STLFSI2	*y*	1.5173	− 0.5598	− 0.9547	0.0001	1.4073	− 0.4105	− 0.3648	− 0.0113

Among the remaining five financial market time series, there are twenty possible couplings. For space reasons, we will only display the most representative ones; the omitted ones follow their behavior closely. In their treatment, we occasionally observed correlations on the residuals, which led us into further investigations on integration, mean reversion, and ARCH effects, suggesting that some financial time series might not properly satisfy the finiteness condition imposed on the first four distribution moments^49^.

### Chaotic or random data?

To check whether expected chaotic aspects are indeed in the data, and if so, how well they are reflected by the model, we followed the standard approach to evaluate for the data and the obtained simulations the maximal Lyapunov exponents (MLE)^2,3,50–55^. The MLE calculated on the first differences of STLFSI2 yields a value of 0.22, to be compared to 0.26 obtained for the Rulkov map. These values are close to those of the logistic map at nonlinearity parameter around 3.6 (chaotic phase), and point at the presence of low-dimensional chaos in the data. The approximate entropy (AE) is used to quantify the amount of regularity and the unpredictability of fluctuations over time-series^56^; applications to financial time series are wide-spread^57–59^. If the value is very small, this implies that the sequence is regular. As an example, for the binary signals S1 = [0 1 0 1 0 1 0 1 0 1 0 1 0 1 0 1] (as a regular alternation of 1 and 0’s) and S2 = [1 1 0 1 1 1 1 0 1 0 1 0 0 0 0 1] (as a part of a random sequence of 0 and 1’s) AE yields 0.0022 and 0.6283, respectively. Applied to the first data differences, the result obtained for STLFSI2 was 0.5045, versus 0.6368 of the Rulkov map modeling.

The correlation fractal dimension *D* is a standard measure of the dimensionality of the space occupied by a set of random points^2,3,60,61^. For the first differences of STLFSI2 and of the Rulkov map, the evaluated correlation dimensions are 4.37 and 4.65. These values are in line with what was reported earlier in Orlando^62,63^, see also^59,64^. Generally, the interpretation of such estimates, must, however, be taken with care, as differencing can be seen as filtering, which will generally affect the results^22,65^. Moreover, a difficulty of the method is to arrive at the required saturation of the curves for increasing embedding dimensions in the presence of noise. Therefore, the exhibited results are more of a corroborating nature. The Hurst exponent (*H*) of single time series is directly related to *D* by the relation $D = 2 - H$^66–68^. For the first difference of STLFSI2 to the corresponding Rulkov map, we found for the *x*-dynamics $H=0.40$ and $H=0.69$ for the *y*-dynamics, respectively. For various time-series, Table 5 lists the MLE, AE, D and H, together with the values obtained from the corresponding Rulkov map approach, evidencing a good agreement between the model and the empirical time-series data properties. For all these computations, the investigated time series were of a sufficient length.

**Table 5:** Chaotic descriptors Maximal Lyapunov Exponent MLE, Correlation Dimension D, Hurst exponent H of financial time series TS (all figures refer to differenced/detrendized data).

Model specification	Real TS	Rulkov map	MLE		AE		D		H	
		State variable	Real TS	Rul. map	Real TS	Rul. map	Real TS	Rul. map	Real TS	Rul. map
Single map	STLFSI2	*x*	0.2214	0.2606	0.5045	0.6368	4.3687	4.5045	0.4015	0.6922
		*y*		0.2604		0.6200		4.0457		0.6294
Single map	SWAP1Y3M	*x*	0.2247	0.2775	0.3488	0.3825	3.5595	3.1941	0.5425	0.6760
		*y*		0.2771		0.3349		3.5939		0.6227
Single map	SPX	*x*	0.1869	0.1672	0.6793	0.9145	4.3073	4.5279	0.4755	0.7305
		*y*		0.1816		0.6878		3.8823		0.5710
Single map	IBOV	*x*	0.1098	0.0917	0.4788	0.4967	2.0135	5.4796	0.7170	0.8207
		*y*		0.1452		0.4603		2.9497		0.8228
Single map	BAMLEM	*x*	0.1152	0.2123	0.3266	0.2953	4.0107	4.0950	0.6563	0.7272
		*y*		0.1488		0.3119		3.4059		0.7078
Single map	DGS10	*x*	0.2674	0.3135	0.6071	0.7537	4.3594	4.1161	0.4551	0.5033
		*y*		0.2759		0.5601		3.5180		0.5292

## Details of model comparison

**Figure 5:**
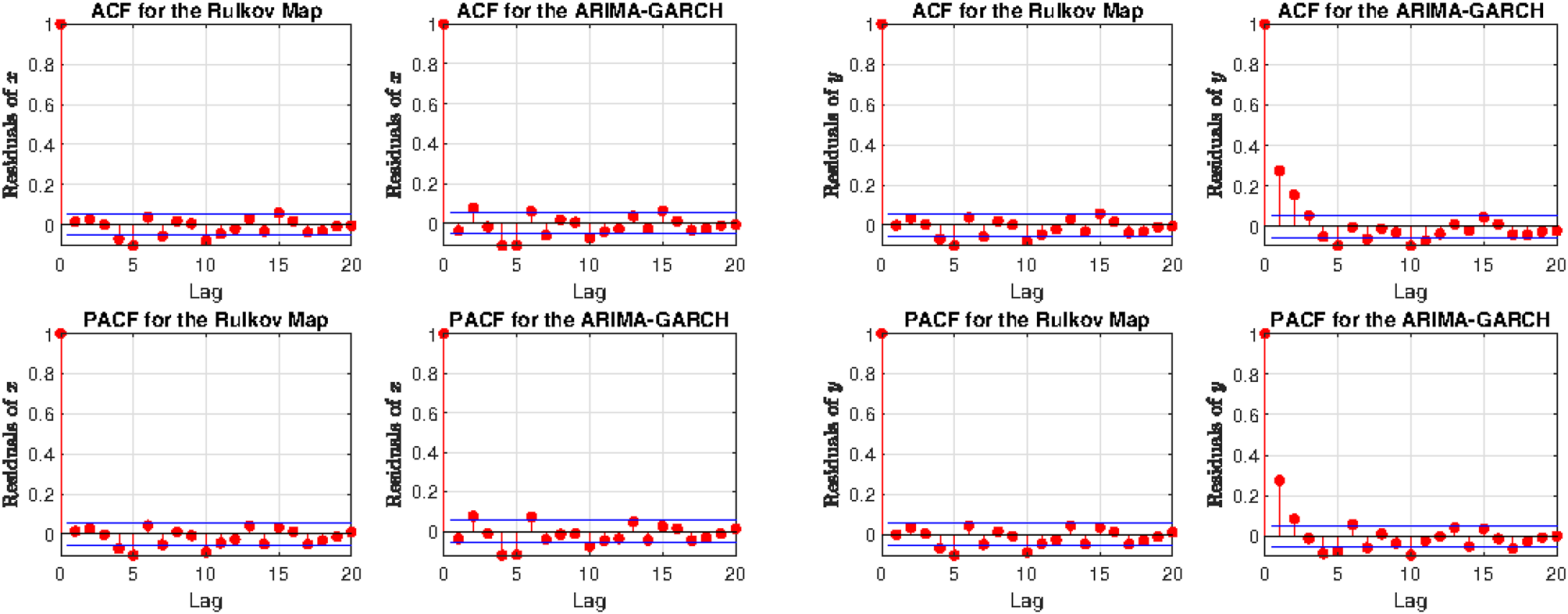
Autocorrelations ACF and partial autocorrelations PACF for state variables *x* (first two columns) and *y* (last two columns), comparing the Rulkov map to ARIMA-GARCH$^*$ (ARIMA(2,1,2)-GARCH(1,1)). While autocorrelation is almost absent in the Rulkov map, the ARIMA-GARCH$^*$ displays significant autocorrelations. This could be taken as an indication of the greater explanatory power of the deterministic approach.

The autocorrelation function (ACF) and the partial autocorrelation function (PACF) on the residuals for state variables *x* and *y* of the Rulkov map and for the ARIMA-GARCH$^*$ model (Fig. 5) indicate that the first order differencing is more effective in removing autocorrelation on the residuals for the Rulkov than for the ARIMA-GARCH$^*$ approach. This could be seen as a sign of the greater explanatory power of the Rulkov map: Although sometimes correlated residuals are features of the research design or intrinsic to the studied phenomenon, “..the inclusion of correlated residuals in latent-variable models is often regarded as a statistical sleight of hand, if not an outright form of cheating”^69^. As STLFSI2 is a combination of other indices and, according to  Lo et al.^70,71^, there is significant evidence of positive serial correlation for weekly and monthly holding-period index returns. Generally, weekly and monthly stock returns are weakly negatively correlated, whilst daily weekly and monthly index returns are positively correlated^72^. This effect has been explained by positive cross-autocorrelations across individual securities across time, where it may happen that this cannot be removed completely^72^. The absence of a unit root, however, has been confirmed using the KPSS test^73^.

**Figure 6:**
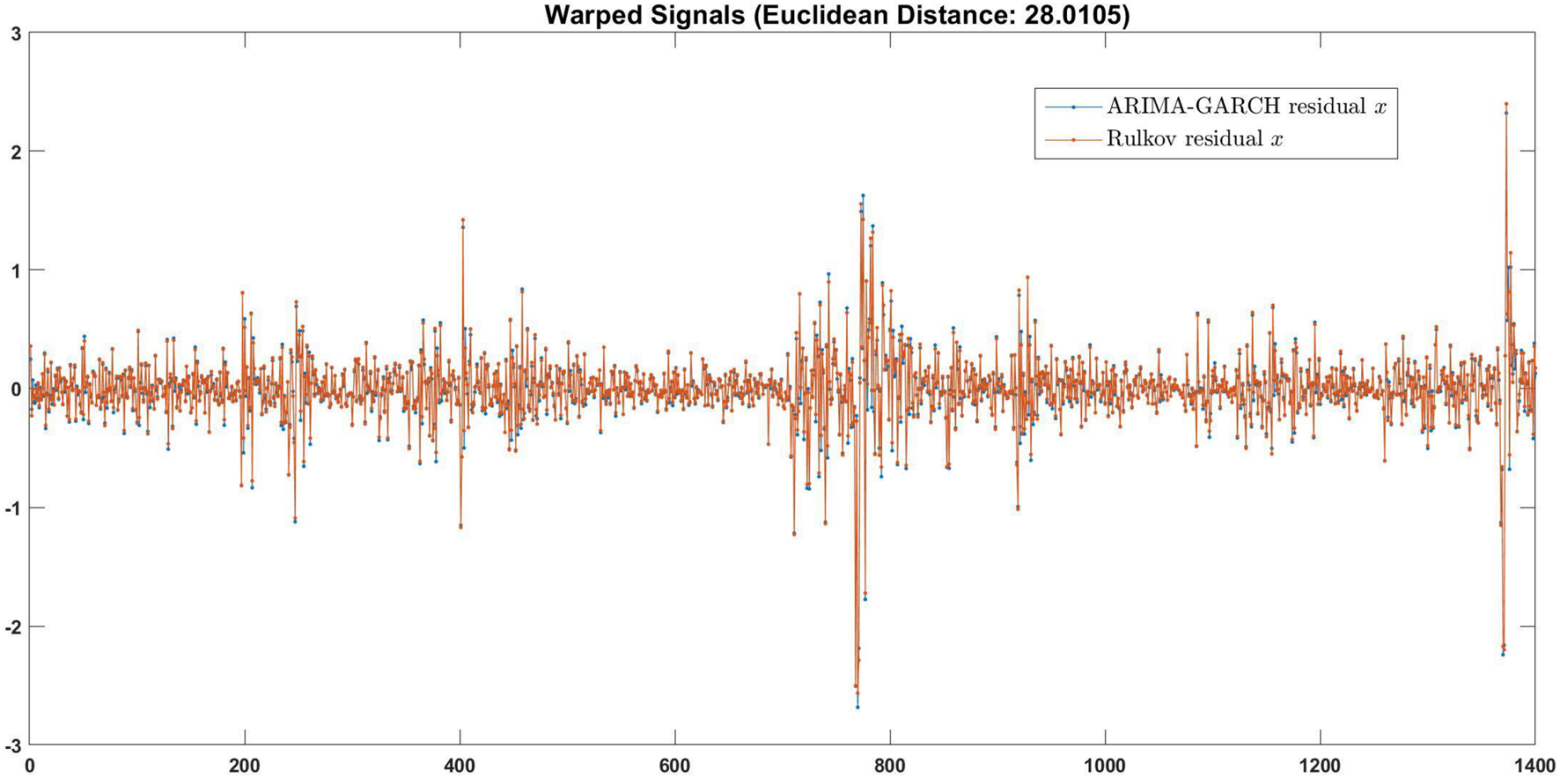
Rulkov map and ARIMA-GARCH$^*$ residuals almost coincide.

To determine how close the Rulkov and the ARIMA-GARCH$^*$ approaches would be, we applied the commonly used the DTWD measure. We first calibrated DTWD at the simple example of a perturbed sawtooth wave that was replicated upon adding Gaussian white noise of variable strength (cf. Fig. 2). Figure 6 shows that the warped residuals of the Rulkov and the ARIMA-GARCH$^*$ *x*-component are almost identical and closely follow the original timeline (in the sense that the warping did not essentially compress or stretch the dependent time series). For the *y* component, we obtained corroborating results (Table 6, corresponding plots are withheld for space reasons). With the only exception of the DTWD for $x^j$ of the IBOV, our distances in Table 6 are below the DTWD generated by Gaussian white noise. From this, we conclude that the distinction between the ARIMA-GARCH$^*$ and the Rulkov map approaches should be of, or even below, the order of the influence of noise.

In Table 6 we report the RMAE accuracy of the Rulkov map, of the Naive, and of the ARIMA-GARCH$^*$ models, the NRMSE of the Rulkov map, of the ARIMA-GARCH$^*$ model, and their DTWD. As may be expected, in all instances the Rulkov map performs substantially better than the Naive model, whereas a comparison between the Rulkov and the ARIMA-GARCH$^*$ yields mixed results: Whereas the Rulkov map’s NMRSE is always lower, its MAE is occasionally higher. The differences are small in all cases, which is corroborated by the residuals’ DTWD.

**Table 6:** Error and DTWD measures of Rulkov map vs. Naive and vs. ARIMA-GARCH* models.

Model specification	ARIMA-GARCH$^*$ parameters	Index	Rul. map state var.	RMAE (Rul. map/ARIMA-GARCH$^*$ )	RMAE (Rul. map/naive)	NRMSE Rul. map	NRMSE ARIMA-GARCH$^*$	DTWD Rul. map vs ARIMA-GARCH$^*$
Single	(2,1,2)-(1,1)	STLFSI2	*x*	0.9880	0.6348	0.0592	0.0598	28.0105
	(2,1,1)-(2,1)		*y*	0.9963	0.8482	0.0484	0.0505	27.0147
Single	(2,0,2)-(2,1)	SWAP1Y3M	*x*	0.9670	0.7971	0.0789	0.0823	24.0586
	(2,0,1)-(2,1)		*y*	0.9911	0.9270	0.0636	0.0641	8.6348
Single	(2,1,2)-(1,1)	SPX	*x*	1.0027	0.7085	0.0702	0.0703	7.1274
	(1,1,2)-(2,1)		*y*	1.0035	0.9060	0.0480	0.0480	2.0015
Single	(1,1,2)-(2,1)	IBOV	*x*	1.0022	0.7039	0.0543	0.0544	5.5793
	(2,1,1)-(2,1)		*y*	0.8255	0.9055	0.0607	0.0706	12.9636
Single	(1,1,1)-(2,1)	BAMLEM	*x*	1.0280	0.8443	0.0651	0.0636	0.9971
	(1,1,1)-(1,1)		*y*	1.0003	0.9236	0.0372	0.0374	0.1439
Single	(1,1,2)-(2,1)	DGS10	*x*	0.9858	0.8001	0.0447	0.0456	6.3229
	(2,1,1)-(2,1)		*y*	0.9957	0.9260	0.0358	0.0357	2.2329
Coupled	(1,0,2)-(2,1)	SWAP1Y3M	$x^i$	0.9915	0.7972	0.0792	0.0854	29.5268
	(2,0,1)-(2,1)		$y^i$	0.9898	0.9268	0.0636	0.0894	8.5407
	(1,1,2)-(2,1)	DSG10	$x^j$	1.0434	0.4940	0.0428	0.0605	59.5394
	(1,1,1)-(2,2)		$y^j$	1.0078	0.6774	0.0533	0.0558	3.1080
Coupled	(2,1,2)-(1,1)	SPX	$x^i$	1.0025	0.6981	0.0661	0.0662	3.6473
	(1,1,2)-(2,1)		$y^i$	1.0029	0.9000	0.0601	0.0706	1.1514
	(2,1,2)-(2,1)	DSG10	$x^j$	0.9834	0.7978	0.0446	0.0453	39.7208
	(1,1,2)-(2,1)		$y^j$	0.9956	0.9259	0.0358	0.0481	2.2309
Coupled	(1,1,1)-(1,1)	SPX	$x^i$	1.0039	0.6735	0.0741	0.0751	2.8845
	(1,1,2)-(1,1)		$y^i$	1.0034	0.8847	0.0651	0.0750	0.8522
	(1,1,2)-(2,1)	IBOV	$x^j$	0.9988	0.7004	0.0542	0.0542	53.1134
	(2,1,2)-(2,1)		$y^j$	0.8262	0.9045	0.0608	0.0730	12.9259
Coupled	(2,0,2)-(2,1)	SWAP1Y3M	$x^i$	0.9721	0.7920	0.0714	0.0740	21.3947
	(2,0,1)-(2,1)		$y^i$	0.9937	0.9307	0.0643	0.0891	7.4690
	(1,1,2)-(2,1)	BAMLEM	$x^j$	1.0495	0.5158	0.0471	0.0619	9.8363
	(2,1,2)-(2,1)		$y^j$	1.0086	0.6814	0.0509	0.0528	0.4621
Coupled	(2,0,1)-(2,1)	SWAP1Y3M	$x^i$	0.9858	0.8032	0.0651	0.0701	21.3392
	(1,0,1)-(2,1)		$y^i$	0.9877	0.9332	0.0613	0.0863	5.5554
	(1,1,1)-(1,1)	IBOV	$x^j$	1.0692	0.4132	0.0416	0.0715	115.3368
	(1,1,2)-(2,1)		$y^j$	1.0081	0.5898	0.0512	0.0555	2.0392

Rulkov map results are slightly superior to those of ARIMA-GARCH*, where DTWD emphasizes the exceptional closeness of the results (all time series were detrendized).

## Forecasting power

**Figure 7:**
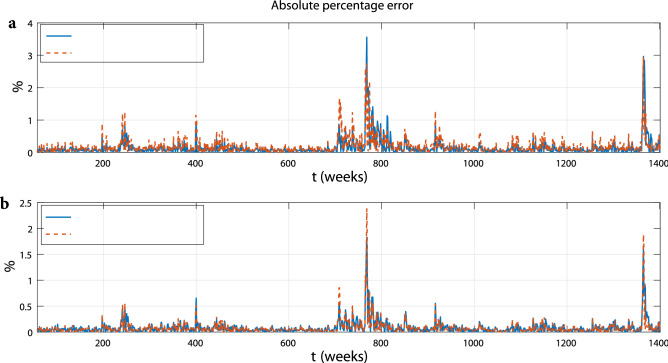
Absolute percentage error between the forecasts produced by the Rulkov map and the ARIMA-GARCH$^*$ model, separately for $x_t$ (plot a) and $y_t$ (plot b).

Of particular interest is that the Rulkov map may easily be used for forecasting purposes. Indeed, once the values $\{x_s,\;y_s\}$ at time *s* are known, the previsions at next time $t>s$ are computed deterministically through Eq. (3). In our set-up the parameters are calibrated over the previous window $[s-m+1,s]$ of size *m*, opportunely chosen. Because we deal with a weekly frequency dataset, we set $m=52$. In particular, starting from *m*, we predict the next values through a rolling window of fixed size *m*. The parameters are calibrated by a least squares regression. We run a robust estimation with the iteratively re-weighted least squares algorithm^34^ which, at each iteration, recomputes the weights based on the residual from the previous iteration. This process progressively continues until the weights converge. Using MAPE, the generated forecasts are compared with those of the ARIMA-GARCH$^*$ model, see Table 7 and Fig. 7. The comparison provides evidence of fits of similar quality, both for the process and its trend, confirming that a deterministic approach performs as well as a stochastic approach.

**Table 7:** MAPE of the Rulkov and the ARIMA-GARCH* model.

MAPE	Rulkov map	ARIMA-GARCH*
$x_t$	0.0396	0.0345
$y_t$	0.0141	0.0152

Finally, we include a summary of results that we have obtained from the application of two more recent approaches to our data. The first is the G2++ model, a popular two-factor Gaussian model where the state process is given by the sum of two correlated Gaussian factors plus a properly chosen to fit real data deterministic function. The model is analytically tractable and its (conditional) distribution, as well as its moments, is given in closed formula. For the aforementioned reasons, Gaussian models like this G2++ model are useful in practice (see^74^, Chapter IV]). The second, the JOU-model, is a (mean-reverting) Ornstein-Uhlenbeck process with Poisson jumps^75,76^. The results in terms of error for the calibrated models with our optimized parameters evidences that in terms of RMAE and DTWD, and MAPE for forecast, the Rulkov map performs better than the two alternatives, although the JOU approach’s results were quite close to the Rulkov and ARIMA-GARCH* results (details of the comparison available upon request from the authors). The calibration time of the JOU approach was roughly thrice that of the Rulkov approach; G2++ required only slightly more time than Rulkov.

## Conclusions

The low DTWD and the almost coinciding residuals of the Rulkov map and ARIMA-GARCH* exhibit a similar quality of the two approaches, indicating that the deterministic chaotic model performs as well as an ultimately refined stochastic one. Furthermore, the low-dimensional deterministic Rulkov map approach suffers less of residuals’ autocorrelation, hosts an improved NRMSE and a better DTW property. A main shortcoming of the ARIMA-GARCH is that generally it does not offer a nice handle towards the understanding of its building and the meaning of the resulting coefficients. The explicitness and simplicity of the Rulkov map opens, in contrast, the door for a deeper understanding of the drivers of the market dynamics. For a full understanding the consequences that a variation of the involved parameters will entrain in the context of the real-world financial markets, a more explicit mapping of the model parameters to financial market parameters will, however, be needed. This would require the building of a kind of conceptual real-world model, helped by the Rulkov equation, but replacing it. Once the essential market parameters would been identified in this way, the degree of precision of such a model can be assessed by using a recent high-level model testing^77^.

Regarding the long-standing debate whether we should consider financial times series as stochastic or rather chaotic^78^, our direct modeling approach yields evidence for a stronger chaotic data component than what one could probably expect. While both elements seem to be present, the chaotic component offers several options for understanding and, finally, monitoring financial processes, if choosing the appropriate time-scale. In particular, our approach may be expected to host an improved explanatory power to events of interest, such as shocks that emerge as endogenous, instead of the exogenous nature supposed by stochastic models^24^. Given this situation, we are happy to show that a substantial contribution of determinism is found in the data^4,79^. For forecasting, the latter is sufficient; the question whether the whole of the data should be termed chaotic or stochastic is, seen in this perspective, of a more ’academic’ nature^1,63^, where the contradicting arguments available may indicate a lack of appropriate technical or theoretical descriptors to deal with such type of data.

More generally, what we deal with here is similar to the one encountered in neuroscience, where initial ideas of simple chaotic or of power-law behavior characterizing the data, had to be replaced by more complicated and, in particular, scale-dependent characterizations^80^. This shifts the question towards determining exactly at what time-scales the two processes of low-dimensional chaos and of stochastic, respectively, are dominant, and to identify the horizon over which a deterministic prediction will yield optimal forecasting results^32^. In this way, our work may open a general avenue towards a better understanding and monitoring of the essential drivers in the data, not only in financial market dynamics, but also in similar processes beyond.
